# Enablers and obstacles for implementing rewilding pathways in socio-ecological systems: Insights from the Oder Delta

**DOI:** 10.1007/s13280-026-02355-5

**Published:** 2026-02-20

**Authors:** Julian R. Massenberg, Stephanie Jahn, Néstor Fernández, Augustin Berghöfer, Christoph Schröter-Schlaack, Birte Kaddatz, Sandeep Sharma, Wiebke Brenner, Bernd Hansjürgens, Erik Aschenbrand, Ulrike Tröger, Ulrich Stöcker, Uta Berghöfer, Johannes Schiller

**Affiliations:** 1 Department of Environmental Science - Environmental Social Science and Geography, Aarhus University, Roskilde, Denmark; 2 Department of Economics, Helmholtz Centre for Environmental Research – UFZ, Leipzig, Germany; 3 Research Unit Environment and Society, Helmholtz Centre for Environmental Research – UFZ, Leipzig, Germany; 4 Department of Environmental Politics, Helmholtz Centre for Environmental Research – UFZ, Leipzig, Germany; 5 German Centre for Integrative Biodiversity Research (iDiv) Halle-Jena-Leipzig, Leipzig, Germany; 6 Institute of Biology, Martin Luther University Halle-Wittemberg, Halle (Saale), Germany; 7 Department of Sustainable Economy, Eberswalde University for Sustainable Development, Eberswalde, Germany; 8 Department of Landscape Management and Nature Conservation, Eberswalde University for Sustainable Development, Eberswalde, Germany; 9 Rewilding Oder Delta e.V., Glashütte, Rothenklempenow, Germany; 10 Wasserwerk der Zukunft e.V., Malchin, Germany

**Keywords:** Biodiversity conservation, Ecological restoration, Multifunctional landscapes, Stakeholder engagement, Transformation

## Abstract

Rewilding gained attention as a transformative approach to restoring biodiversity and ecosystem functions in Europe’s multifunctional landscapes. Yet, its implementation in socio-ecological systems (SES) raises critical questions about the alignment of ecological goals with social values, cultural contexts, and economic realities. This article discusses enablers and obstacles for rewilding in human-shaped landscapes, drawing on a holistic, transdisciplinary analytical frame that integrates ecological, economic, and social dimensions. Using the Oder Delta as an illustrative case study, we discuss enablers and obstacles that shape rewilding pathways, including ecological factors but also inclusive processes, value pluralism, and institutional coordination. Based on the case-based results, our discussion offers insights on how rewilding could serve as a complementary strategy to established nature conservation. By situating rewilding within the complexity of SES, this article contributes to an emerging discourse on inclusive landscape restoration and the future of biodiversity governance, offering transferable insights for policy and practice.

## Introduction

Despite recent efforts and achievements in biodiversity protection, overall biodiversity continues to decline under many different land uses (IPBES 2018), including in protected areas (see e.g., Hallmann et al. 2017) and aquatic ecosystems (Haase et al. 2025). While protected areas remain important for conservation efforts, their further expansion is challenging (Le Saout et al. 2013). In many regions, particularly in Europe, protected areas coexist with productive landscapes where conservation objectives must be integrated into multifunctional landscapes. Consequently, effective biodiversity conservation builds on complementary approaches that operate beyond designated areas and focus on the scaling up of ecological restoration across multiple land uses, including agricultural and forested landscapes (Grass et al. 2021).

Against this background, rewilding offers a narrative that links ecological restoration and biodiversity conservation with society and local economic development (Jepson et al. 2018; Jepson 2019). Recent work emphasizes that rewilding and ecological restoration pursue complementary yet distinct goals, with rewilding focusing more strongly on functional ecosystem processes and nature-led dynamics, while restoration traditionally emphasizes compositional goals and predefined outcomes (see Carver et al. 2025). Rather than pursuing wilderness or focusing solely on land abandonment, rewilding focuses on *increasing wildness*, i.e. restoring the autonomy of natural processes and self-sustaining ecosystems (see, e.g., Fernández et al. 2017; Perino et al. 2019; Carver et al. 2021; Massenberg et al. 2023). This distinction is crucial in order to explicitly recognize the socio-economic and socio-cultural contexts in landscapes as well as biodiversity restoration (see, e.g., Massenberg et al. 2023). Rewilding should pursue synergies between more self-sustaining and functionally resilient ecosystems and local economies, thus fostering an environment where biodiversity recovery goes hand in hand with community development. By focusing on restoring ecological processes, landscape connectivity, and biodiversity, rewilding may also generate a wide range of co-benefits for local communities and local economic development, including opportunities in nature-based tourism, sustainable agriculture and forestry (Jepson et al. 2018; Massenberg et al. 2023; Faure et al. 2024).

Implementing rewilding is particularly complex in densely populated and multifunctional landscapes such as those found in Europe, where ecological, economic, and social interests intersect. These landscapes can be understood as social-ecological systems (SES) which are shaped by historically grown interactions of ecological, economic, social, and cultural factors. Typically forming mosaics of diverse land uses and management intensities, SES face multiple, often conflicting, demands (Bennett et al. 2006; Lorimer et al. 2015; Semenchuk et al. 2022).

The Oder Delta exemplifies these dynamics. Located on the German–Polish border, it forms a mosaic of agricultural land, grassland, forests, wetlands, and estuarine ecosystems shaped by centuries of human activity. The region is one of Rewilding Europe’s ten priority areas and serves as a living laboratory for exploring how rewilding can be implemented in SES. Motivated by the re-establishment of natural hydrological and trophic processes, the enhancement of biodiversity, and the creation of sustainable livelihoods, rewilding efforts in the Oder Delta were initiated by the cross-border NGO Rewilding Oder Delta e.V. (hereafter, ROD). Drawing on research conducted within the REWILD_DE project “Sustaining Biodiversity and Creating Ecosystem Service Opportunities through Rewilding—Learning from the Oder Delta” this paper examines the enablers and obstacles to implementing rewilding within SES. The aim is to develop a holistic rewilding pathway that captures the interconnections between ecological, economic, and social dimensions and explores how rewilding can be integrated into SES such as the Oder Delta. Our findings from the Oder Delta emphasize how socio-ecological complexities and local conditions shape rewilding strategies. Thus, while rooted in the specific context of the Oder Delta, our findings offer valuable lessons for other rewilding efforts in Germany as well as across Europe and hold strong potential for transfer and mutual learning within the broader rewilding network.

The following “Setting up a holistic rewilding pathway: Key questions” section outlines the key research questions and methodological approaches that underpin the development of this holistic rewilding pathway. “Three analytical building blocks for a holistic rewilding pathway” section introduces the three analytical building blocks—‘ecological dimension’, ‘economic dimension’, and ‘social dimension’—which structure the paper’s core analysis. These perspectives will first be outlined in detail and then brought together in a case-specific rewilding pathway that integrates their interrelations presented in “Rewilding pathways: Embracing the complexity of ecological, economic, and social contexts” section. This integrative perspective provides the foundation for the subsequent discussion and concluding reflections in “Discussion and outlook” section.

## Setting up a holistic rewilding pathway: Key questions

Setting up a holistic rewilding pathway involved addressing key research questions concerning the implementation of rewilding in SES. Building on the research gaps identified by Massenberg et al. (2023), these questions reflect persistent challenges in rewilding research and practice—including the need for clearer and context-sensitive definitions of rewilding in multifunctional landscapes, improved integration of inter- and transdisciplinary knowledge, a better understanding of value pluralism and local perceptions, and the development of suitable indicators and governance frameworks for assessing rewilding progress. Table 1 provides an overview of these research questions, summarizing how some of these were addressed in the REWILD_DE project and which methodological approaches were applied to explore how rewilding can be integrated into local realities and to offer insights for broader applications.

**Table 1:** Addressing research gaps in rewilding: focus areas and methodological approaches applied in the REWILD_DE project fostering a holistic understanding of rewilding (*Source*: Own creation based on Massenberg et al. (2023))

Research area	Topic	Research gap	Methodological approach
What is the goal of rewilding?	Conceptualization and definitions of rewilding	Need for clear and context-sensitive definitions of rewilding in human-dominated and multifunctional landscapes	Literature review, elicitation of stakeholders’ expertise
What is the rewilding potential for reconciling nature conservation and regional development?	Regional context analysis and identity	Understanding the role of place-based identity in supporting/opposing rewilding views. Alignment with local economic strategies	Interviews with local stakeholders, participatory workshops, rewilding pathways (scenario development)
	Regional economic development potential	Understanding the economic opportunities and challenges of rewilding for local enterprises as well as incentives and barriers for rewilding implementation	Surveys and interviews with local enterprises, analysis of regional development strategies
What are inhabitants’ preferences, values, and perspectives?	Values of nature and landscapes	Understanding the pluralism of values related to rewilding and existing land use, as well as public support for rewilding	(Deliberative) Discrete choice experiments (DCEs) and surveys on participants' underlying motivations, scenario development, narrative interviews
How is rewilding embedded in multifunctional landscapes?	Scale of analysis	Examining the role of network effects in multifunctional landscapes and the interplay between protection and land uses	Historical landscape analysis, ecological monitoring, scenario development
How can rewilding effectiveness be evaluated?	Monitoring and ecological change assessment	Understanding how rewilding influences ecosystem composition, structure, and functions	Ecological monitoring, scenario development

**Fig. 1:**
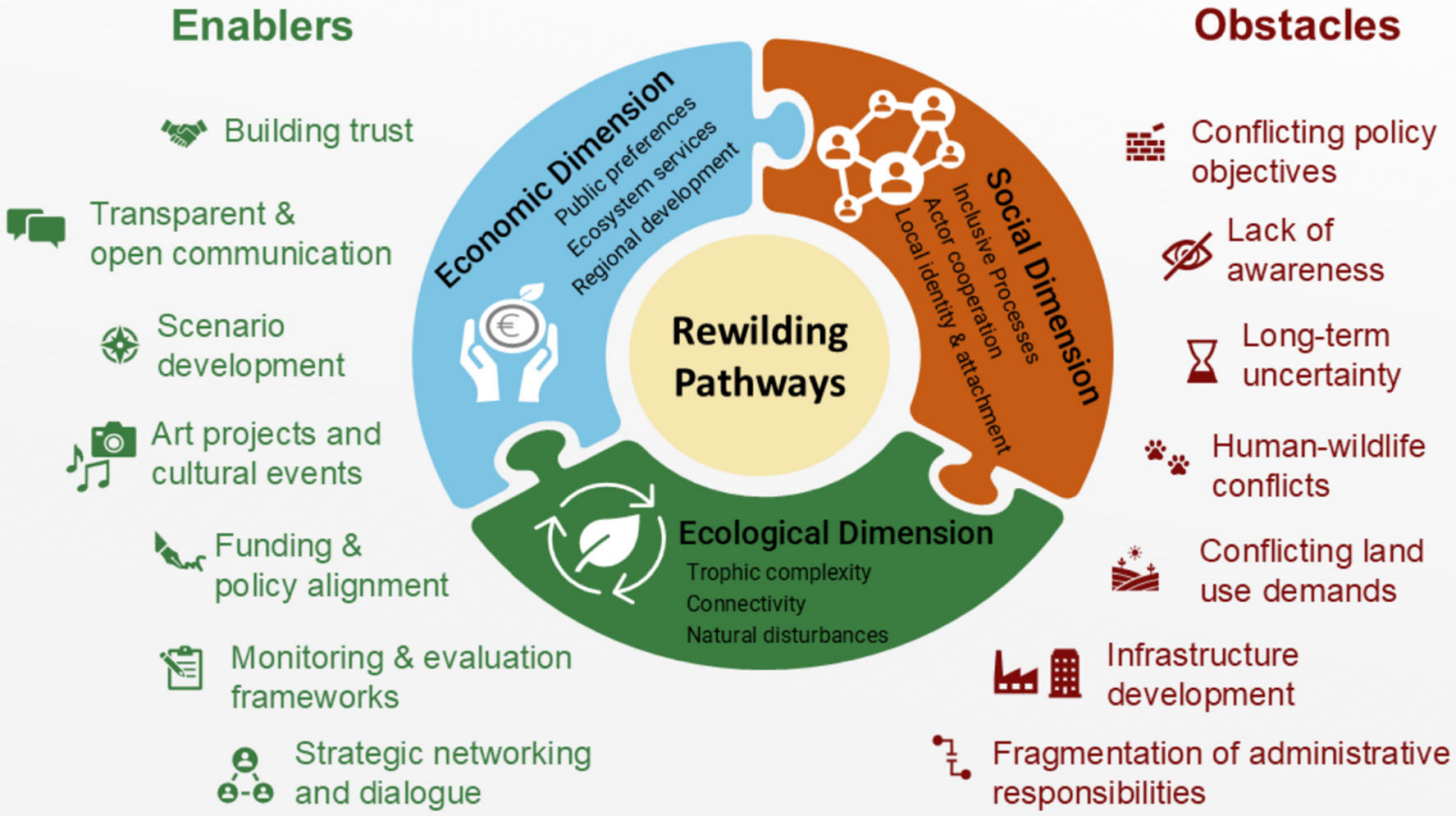
Analytical building blocks and associated enablers and obstacles for the implementation of rewilding pathways. The listed enablers and obstacles are based on empirical findings from the REWILD_DE project

These research questions highlight that successful rewilding requires an integrated understanding of ecological, economic, and social processes and their interactions. Three interrelated components and associated perspectives were identified as critical for understanding the complexities of rewilding efforts in SES, such as the Oder Delta: ‘ecological dimension’, ‘economic dimension’, and ‘social dimension’ (see Fig. 1). Although these dimensions are not entirely distinct, distinguishing them helps as focal points when developing a rewilding pathway. In practice, many elements span multiple dimensions. For example, ecosystem services represent an anthropocentric concept that links ecological processes with social and economic benefits (see, e.g., de Groot et al. 2002), thus, they could be situated across all three dimensions. Similarly, public preferences, depending on whether they are framed in economic or social terms, reflect both individual valuation and collective perceptions. Acknowledging these overlaps underscores the integrated nature of socio-ecological systems, where ecological functions, economic incentives, and social values are interrelated rather than operate in isolation. For analytical clarity, we treat these intertwined dimensions as analytical building blocks of rewilding pathways, which together capture the diverse enablers and obstacles influencing rewilding outcomes. In the context of this study, enablers refer to factors that facilitate or support rewilding implementation while obstacles denote barriers that hinder rewilding progress. Figure 1 illustrates these three dimensions together with the associated obstacles and enablers for a successful rewilding pathway. They are discussed in detail in the following section.

## Three analytical building blocks for a holistic rewilding pathway

For successfully setting up a holistic rewilding pathway, the three analytical building blocks (Fig. 1) have to be dealt with. Failure to do so may result in significant obstacles to rewilding activities. In the following, we discuss these building blocks in detail including the potential obstacles and how they can be weakened or overcome.

### Ecological dimension

Rewilding approaches ecological restoration from a complex-systems perspective, based on the premise that self-sustaining ecosystems with greater complexity harbor higher biodiversity value and confer increased resilience to change (see, e.g., Perino et al. 2019). Unlike restoration approaches focused on recreating native reference baselines, rewilding strives to minimize human control and promote the autonomy of natural processes to drive restoration. The restoration of ecosystem processes and functions is central to ecological rewilding, with a focus on increasing trophic interactions and functions, facilitating natural recolonisations with minimal human interference (Fernández et al. 2017; Pereira et al. 2024), reducing barriers to species dispersal, and reinstating natural disturbance regimes (Torres et al. 2018; Perino et al. 2019). Rewilding in human-dominated landscapes poses major challenges, as land management often suppresses natural disturbances, increases fragmentation and barriers to species movement, and leads to the extirpation of species with key functional roles, such as large predators and herbivores.

Although rewilding does not aim to replicate historical ecological conditions, historical ecology provides valuable insights into the anthropogenic drivers underlying the current ecosystem state (Turvey and McClune 2025). Such information can support the design of rewilding strategies that link past trends with future scenarios. The Oder Delta, a lowland region currently characterized by forestry, agriculture, and pasture, was historically shaped by flooding regimes within a complex mosaic of hydrological formations. Large-scale human modifications of its hydromorphology have intensified since the late Medieval period, resulting in drastic reductions of floodplains and peatlands and their replacement by farmland (Kaiser et al. 2012). However, forest cover has remained relatively stable since the eighteenth century (Sharma et al. in review), consistent with recent trends across Europe (Kaplan et al. 2009), although increased patch fragmentation and widespread substitution of broadleaf forests with conifer monocultures have been observed. Finally, the large-mammal trophic complexity has been gradually reduced, beginning with the extinction of all three megafauna herbivores by the sixteenth century—Auroch (*Bos primigenius*), European bison (*Bison bonasus*) and Elk (*Alces alces*)—followed by the loss of large carnivores—the bear (*Ursus arctos*), wolf (*Canis lupus*) and Eurasian lynx (*Lynx lynx*)—in the 18th–nineteenth centuries (Sharma et al. in review).

Elicitation studies involving diverse stakeholder groups identified key rewilding priorities for the region, each contributing to the restoration of essential ecosystem functions and services: (1) enabling the comeback of large predators and herbivores while restoring degraded trophic functions; (2) reinstating natural water dynamics and flooding regulation across agro-ecosystems; and (3) enhancing connectivity, habitat heterogeneity, and the biodiversity value of agricultural and forest landscapes (Segar et al. 2022b; Quintero-Uribe et al. in review). Building on these priorities, Segar et al. (2022a, b) assessed 19 indicators of rewilding in the region and documented moderate progress over the last decade. This positive trend largely reflected increasing large herbivore abundances and the recolonization in recent decades of keystone species such as wolf and beaver (*Castor fiber*), both of which have established viable populations in the region (Neubert and Wachlin 2004; Jarausch et al. 2021). While trophic rewilding effects remain to be tested in the Oder Delta, positive regulatory functions of wildlife comebacks can be anticipated.

The recolonizing wolf population may provide an effective, nature-based means of controlling high-impact wildlife diseases, such as African swine fever (ASF), by helping suppress disease emergence and reduce prevalence. The rising incidence of African swine fever in wild boar populations in neighboring Poland, combined with recent detections in Germany (Sauter-Louis et al. 2021), has heightened concern about wildlife-to-livestock transmission (Fleischmann 2025). This situation has led to the implementation of intensive control measures, including the erection of a fence along the German–Polish border—a measure of questionable effectiveness with the potential to cause far-reaching ecological consequences (Bhardwaj and Selva 2025). Importantly, models of the ASF–wild boar system indicate that wolf consumption of infected carcasses could reduce the severity of ASF outbreaks (O’Neill et al. 2020), highlighting a largely overlooked role of wolf scavenging in wildlife disease management strategies (Sauter-Louis et al. 2021). Further progress will be also required to restore higher integrity trophic functions, a core principle of rewilding (Carver et al. 2021). For example, the potential recolonization of large herbivores like elk and bison from nearby populations in Poland can contribute to generate mosaic landscapes in forest ecosystems, contributing to a mix of forest and open spaces and providing habitat for a variety of species (Pringle et al. 2023). Furthermore, reinstating browsing and grazing functions through ecologically meaningful large-herbivore populations is increasingly regarded as a synergistic climate–biodiversity solution, with substantial potential to enhance ecosystem carbon storage and contribute to climate-change mitigation (Schmitz et al. 2023). At the same time, it can benefit the conservation of threatened and range-restricted plant species in native communities, while limiting the spread of non-native species (Segar et al. 2022a). Despite opportunities for herbivore trophic rewilding in the Oder Delta, significant barriers to movement may hinder their dispersal (Skorupski et al. 2022; Bluhm et al. 2023) and the establishment of self-sustaining populations with meaningful trophic effects on ecosystems.

Rewilding plans in the Oder Delta aim to reinstate natural water dynamics by targeting 200 km of streams and rivers flowing into the Szczecin Lagoon and the lower Oder River, including the removal of barriers to longitudinal, lateral, and horizontal connectivity, thereby enhancing wetland connectivity, water flow, and water retention in agroecosystems through natural processes. At the same time, the recolonization of the Eurasian beaver provides a complementary, self-sustaining form of ecosystem engineering, supporting key hydrological functions such as groundwater retention, drought resilience, flood mitigation, and carbon storage (Wohl 2013; Brazier et al. 2021), all of which align closely with key restoration priorities identified by stakeholders in the region (Quintero-Uribe et al. in review).

Finding opportunities to enhance connectivity and reinstate natural ecological processes in agricultural land will be critical to advancing rewilding objectives in the Oder Delta. Although the transition toward a more nature-based agroeconomy offers opportunities for implementing rewilding projects, this has yet to translate into effective measures that reduce ecological barriers to species dispersal and restore flood regimes in the heavily transformed floodplains (Segar et al. 2022b). For instance, rewilding set-aside land and adopting alternative management approaches within the agricultural matrix—such as permaculture or conservation agriculture, or paludiculture (Tanneberger et al. 2022)—hold unexploited potential for creating wilder, more resilient ecosystems in used landscapes (Rey Benayas et al. 2025). Notably, stakeholders have identified a pressing need to restore natural flood dynamics in the Oder Delta to protect agricultural fields from climate change (Quintero-Uribe et al. in review), a measure that could simultaneously enhance ecological connectivity across the freshwater–terrestrial interface and increase the climate resilience of agroecosystems.

While the restoration of natural processes is a critical pillar of rewilding, ecological goals need to be addressed together with the social and economic opportunities and constraints through integrated socio-ecological perspectives. Rewilding requires the effective management of human-wildlife conflicts, particularly those involving large mammal species. A nationally-representative sampling study in Germany and Poland on public preferences toward rewilding in the Oder Delta revealed strong support for the functional restoration of large herbivores and predators through promoting the comeback of European bison, elk, wolf, and Eurasian lynx (Dunn-Capper et al. 2024). However, this support was significantly lower among respondents living in the region, suggesting that wildlife increases may be perceived as detrimental among rural populations, potentially hindering rewilding efforts (see also Massenberg 2025). Although wildlife comeback is often assumed to increase conflicts with farming, the severity of such conflicts varies across landscapes and regions, and can be significantly reduced through prevention-based policies and appropriate compensation schemes (Bautista et al. 2019). Ecological rewilding should be supported by predictive ecological modeling that helps identify the spatial incidence of conflicts associated with the recovery of large predators and herbivores (Wilkinson et al. 2020). These approaches enable risk identification and the adoption of measures to increase coexistence (e.g., Bautista et al. 2021). Rewilding organisations can play a pivotal role in assisting the implementation of prevention measures, facilitating the dialogue between local communities and policymakers, and developing adaptive compensation schemes (König et al. 2020).

### Economic dimension

The targeted ecological interventions through rewilding discussed above, such as creation of a mosaic landscape with open areas, forests, and restored peatlands that facilitate wildlife comeback, must be accompanied by new economic opportunities to ensure broad support for rewilding. Understanding the economic implications of rewilding, alongside ecological restoration, is essential for envisioning landscapes that are not only biodiverse but also economically viable and socially desirable for local communities. In this sense, the economic perspective highlights rewilding as a multifaceted approach that may contribute not only to ecological restoration but also to local and regional economic development (see, e.g., Pettorelli et al. 2018; Carver et al. 2021, 2025; Convery et al. 2025). These broad economic implications become tangible through concrete opportunities and trade-offs with respect to regional economic development: new income opportunities from nature-based tourism (Faure et al. 2024), ecosystem services such as carbon sequestration (Navarro and Pereira 2012; Schmitz et al. 2023), pollination (Garrido et al. 2019), and extensive land management practices, such as paludiculture (i.e. cultivating specific plants on rewetted peatlands) (Rowan et al. 2022). Comparable developments are evident elsewhere. In the Białowieża Forest, the European bison attracts high visitor numbers and has become a central aspect of regional tourism (Jalinik and Łuniewski 2024), while in the Romanian Carpathians, local residents largely anticipate tourism and income benefits from ongoing bison reintroduction efforts, despite concerns about potential conflicts and land-use restrictions (Vasile 2018). At the same time, in European landscapes shaped by centuries of human activity, such opportunities may come along with trade-offs involving cultural heritage, landscape aesthetics and potential loss of income associated with current forms of land-use change (Höchtl et al. 2005; Drenthen 2018a) as well as human-wildlife conflicts (Perino et al. 2019). Thus, while potentially enhancing biodiversity, long-term ecological resilience, as well as creating new income opportunities, these transformations may also lead to tensions, highlighting the need for approaches that account for economic value, societal values, as well as societal concerns (Schulte to Bühne et al. 2022; Massenberg et al. 2023).

These opportunities and trade-offs also underline how strongly the economic dimension is intertwined with the ecological and social dimensions. Functioning ecosystems underpin benefits such as ecotourism, ecosystem services, and sustainable land management, while social acceptance and cooperation determine whether these benefits can be realized and maintained. At the same time, economic expectations feed back into ecological and social outcomes, for example, when the marketing of charismatic species or landscapes aesthetics shapes conservation priorities or public perceptions of value.

To examine the economic expectations and public perceptions of rewilding in the Oder Delta, several surveys were conducted among local enterprises and the local population. Among these, several stated-preference studies eliciting among others willingness to pay (WTP), were conducted. The findings indicate a general WTP for increased “wildness”, with strong preferences for a more diverse landscape composition, micro-rewilding and the introduction of large herbivores. However, the valuation of heterogeneous landscape composition followed a nonlinear trend, indicating that some respondents preferred medium-sized changes over radical transformations. Furthermore, the presence of wolves, who reappeared in the area, is often viewed negatively, highlighting the complexity of public attitudes toward specific aspects of rewilding (see Massenberg 2025 for details; see also Dunn-Capper et al. 2024).

Furthermore, while many respondents value the ecological aspects of rewilding, there is skepticism toward the economic benefits, particularly those related to regional development through nature-based tourism. The perception of over-tourism, already present in the coastal areas of the Oder Delta, particularly the island of Usedom, one of the most highly frequented tourist regions in Germany, might overshadow potential local economic gains. Additionally, protesters expressed distrust in the economic mechanisms supporting rewilding, highlighting the need for transparent and inclusive policy-making processes (see Massenberg 2025, for details).

Values and beliefs play a crucial role in shaping public attitudes toward rewilding (see, e.g., Carver et al. 2021; Ferraro and Whitehead 2025). Exploring public motivations for supporting or opposing rewilding in the Oder Delta revealed a wide range of values and beliefs. Motivations extend beyond purely instrumental or individualistic considerations, including non-instrumental values (e.g. nature having a value independent of humans), societal importance (e.g. fairness toward future generations), and both consumer and citizen perspectives. For example, many participants were motivated by the idea that nature has a right to exist, by concerns about ecological fragility and/or by emotional ties to local landscapes and cultural identity. These nuanced public preferences and underlying motivations are crucial for designing effective rewilding initiatives and policies. Policies targeting non-heterogeneous attributes (attributes with more consistent public support, such as in this case landscape heterogeneity) may be easier to implement with more predictable public support, whereas attributes with heterogeneous preferences, such as the presence of large predators, may require tailored, localized approaches to address diverse perceptions, preferences, and values (see Massenberg 2025).

With regard to regional enterprises in the Oder Delta region, a survey of their economic potential reveals that the vast majority of the companies surveyed are very open to a “wilder landscape” (Kaddatz and Schulz unpublished manuscript). The economic potential seen by them in connection with rewilding includes increased tourism demand away from the current hotspots in the coastal inland, positive benefits for marketing and attracting new customer groups and/ or markets. Additionally, the potential for enhancing the region’s image was frequently mentioned. This suggests that developing a cross-border regional brand “Oder Delta”, which integrates tourism and regional development around nature and sustainability could be a viable management approach. However, several obstacles need to be overcome in order to leverage the positive attitude of the enterprises. It was noted that parts of the region are currently promoted by different tourism organisations, which hampers the creation of a unified external image (Kaddatz and Schulz unpublished manuscript).

For a smaller percentage of the surveyed companies (18%), there is no visible potential arising from rewilding. This could be attributed to a lack of awareness of rewilding in general and the regional rewilding organization in particular, but also due to the lack of visible examples of economically viable “rewilding enterprises” in the region (Kaddatz and Schulz unpublished manuscript). In agriculture and forestry, one of the challenges lies in addressing the financial implications of reduced agricultural subsidies and potentially lower yields resulting from land conversion, which can make the transition to rewilding or more sustainable land management practices difficult.

Interviews with local enterprises revealed that while some enterprises are not opposed to transitioning to more ecological business models, they require targeted operational advice tailored to their sector, financial compensation, initial investments, and networking opportunities (also across sectors) to achieve this (Kaddatz and Schulz unpublished manuscript). This underscores the importance of creating economic opportunities within rewilding processes.

The findings from the Oder Delta indicate that the economic dimension of rewilding is perceived through diverse and sometimes conflicting perspectives by both local residents and regional enterprises. These perceptions reveal enablers such as generally positive attitudes toward rewilding, interest in tourism diversification and regional branding, and openness to more sustainable business ideas. At the same time, obstacles include constraints to financing, fragmented regional promotion, and limited access to advisory services. Moreover, the inherent uncertainties and long-term nature of rewilding projects complicate their economic valuation and planning. Future research should address these uncertainties, explore public and business preferences over time, and consider inclusive policy-making approaches that incorporate local and scientific knowledge. This may help in balancing local concerns with broader ecological and economic benefits, ensuring the sustainability and acceptance of rewilding efforts.

### Social dimension

Restoration practices are inherently social processes (Drouilly and O’Riain 2021). Accordingly, the concept of rewilding is interpreted and debated by social actors who eventually take action to initiate rewilding processes they consider as beneficial for themselves and the environment (Martin et al. 2021). Major social obstacles include a general lack of public awareness and skepticism toward nature restoration projects, often linked to previous negative experiences or existing conflicts (Wynne-Jones et al. 2018; Butler et al. 2019). In addition, conflicting policy objectives, competing land-use demands (Sandom et al. 2019), and potential human–wildlife conflicts (Perino et al. 2019) further complicate implementation efforts. The fragmentation of administrative responsibilities and decision-making authority across different governance levels and sectors also hampers coherent and coordinated rewilding initiatives (Butler et al. 2021; Martin et al. 2023).

As rewilding represents a voluntary approach to ecological restoration, the success of rewilding projects often depends on local community involvement, deliberation, and cooperation (Carver et al. 2021). Participatory processes can influence both ecological outcomes (e.g., by ensuring biodiversity-friendly land management, reducing human-wildlife conflicts) and economic outcomes (e.g., through local support for nature-based tourism). Social cooperation thus links ecological restoration efforts with economic opportunities discussed in the previous sections, while also shaping landscape perception and local place identity (Drenthen 2018b; Hawkins et al. 2025). Beyond this instrumental perspective on the importance of stakeholder participation in rewilding, the social dimension also embodies a core principle of holistic rewilding: People are an integral part of most landscapes, and rewilding should therefore also comprise human relations with their land (Carver et al. 2021; Ferraro and Whitehead 2025).

To address these challenges in the Oder Delta, the regional NGO ROD developed the “area-focused management” approach, which describes a process in which all relevant actors in the respective sectors (e.g. private sector, administration, associations, NGOs, wider public, etc.) and on the required governance levels (from local to international) collaborate within a clearly defined area (Jahn and Götz-Schlingmann 2025). This approach is based on a thorough analysis of actor dynamics (Reed et al. 2025) and transparent communication, with open exchanges about rewilding sites and their broader socio-ecological benefits at the core. Furthermore, developing shared goals and strategies while facilitating actor dialogue ensures that diverse interests are effectively coordinated (Martin et al. 2023). Focusing on specific delineated areas helps to maintain continuity even if stakeholders and conditions evolve over time. However, the area-based management approach remains embedded within a broader landscape perspective, which must always be taken into account. Therefore, the NGO ROD oversees and coordinates the various individual rewilding sites, ensuring their interaction and coherence, and maintains communication and exchange with actors at higher governance levels who operate at the landscape scale.

In the REWILD_DE project, the targeted processes to establish rewilding activities through the “area-focused management” were supported by, three participatory, community-focused events that helped setting the tone for dialogue in the area, fostering a shared language, bridging perspectives, and encouraging reflection on landscape perceptions (Berghöfer 2025; Berghöfer and Tröger 2025): Landscape walks enabled participants from various professional backgrounds to exchange views while physically moving through the landscape. This allowed participants to set aside professional roles and past conflicts. A music recital and a photo exhibition provided space for residents to express their memories, worries, and dreams, contributing to a broader dialogue about the landscape and its future development. These events created valuable opportunities for dialogue and allowed participants to reimagine the future of their landscapes (cf. “Rewilding pathways: Embracing the complexity of ecological, economic, and social contexts” section).

ROD’s engagement is an illustrative example of how non-profit organisations, especially those driven by environmental values and public interest, can play a crucial role in environmental decision making processes (Newig et al. 2023)—in this case by initiating rewilding efforts, particularly in a region where biodiversity restoration strategies face skepticism. ROD operates without a formal mandate over local actors, but by bringing people carefully in conversation and keeping the process open-ended, they encourage broader participation (Ferraro and Whitehead 2025). Building such networks and mediating interests between various actors is a necessary and highly time-consuming process, requiring sustained effort over many years (Ferraro and Whitehead 2025). Of course, the impacts of such participatory processes largely depend on local community contexts. Hence, ROD fosters the transfer and exchange of experiences from the Oder Delta, enabling mutual learning among regions and supporting the integration of regional insights into the wider Rewilding Europe network^1^.

Taking into account the three building blocks discussed above—the ecological, economic, and social—rewilding benefits from a holistic approach that seeks to integrate these perspectives. Rather than focusing on time-bound interventions or isolated measures within a single field, such an approach aims to develop long-term, interconnected pathways that gradually transform land use and restoration regimes. This perspective emphasizes rewilding as a dynamic and adaptive process of change rather than a fixed end state. How such an integrated pathway can be initiated and tested in practice has been explored within the REWILD_DE project.

## Rewilding pathways: Embracing the complexity of ecological, economic, and social contexts

We use the notion of ‘rewilding pathways’ to describe a structured approach to translating rewilding principles into practice within complex socio-ecological systems. Such pathways are not predefined blueprints but rather open-ended processes that connect ecological, economic, and social dimensions through shared learning, visioning, and experimentation. By engaging local actors, pathways integrate diverse forms of knowledge, values, and expectations into coherent narratives about landscape change. Within the REWILD_DE project, one such pathway-process was initiated in the Oder Delta’s village of Rothenklempenow.

Rothenklempenow is a rural village with around 600 inhabitants. The surrounding landscape features small lakes, beech, and mixed forests, pastures, and fields. Since 2013, local agriculture has largely followed organic and biodynamic standards, supported by a cooperative farming initiative under a land stewardship network. Since 2017, the site has hosted sustainability projects and events, steered by an educational center recognized by UNESCO. Furthermore, the site serves as the headquarters of ROD. Developing rewilding pathways for Rothenklempenow benefited from communication on equal footing, addressing local power imbalances and ensuring that all voices—from experiential to scientific—were heard and valued (cf. “Social dimension” section).

The starting point of this process was not a specific vision for a rewilded landscape around the village, but the question: What matters most to local inhabitants today, and how might rewilding approaches respond to these concerns? Instead of prescribing outcomes, the process began with an open and exploratory scoping phase, combining individual interviews, group discussions and joint field explorations of the surrounding landscape. These encounters helped identify shared challenges and opportunities related to land use, water management, and the long-term prospects of local agriculture and community life.

One key topic concerned the exploration of plausible futures for Rothenklempenow as a living and economic space by 2035. While this broad question may be unconventional for a restoration project, it helped to consider potential rewilding futures from a local perspective, and as part of a broader set of factors shaping landscapes. In response, landscape scenarios for 2035 were co-developed by an interdisciplinary team and local actors (Berghöfer et al. 2024). These scenarios illustrated alternative development trajectories under different conservation and land-use regimes, helping to visualize potential trade-offs and synergies between ecological resilience, economic viability, and social well-being.

The pathway process highlighted that strengthening landscape functions will become increasingly important within conservation and restoration measures, including rewilding, to support resilient, economically viable, and attractive landscapes. Defining priorities for such landscapes is a societal rather than purely technical task that requires inclusive regional dialogue. Yet, effective negotiation formats and instruments for multifunctional land management remain limited. Current agricultural policies often create counterincentives, underscoring the need for conservation and farming actors to jointly advocate for greater regional flexibility and long-term perspectives that benefit both landscapes and rural livelihoods (Berghöfer et al. 2024).

The concept of rewilding pathways (see again Fig. 1) thus captures the interplay of enablers and obstacles encountered throughout the REWILD_DE process. The Rothenklempenow case shows that rewilding evolves not from predefined conservation targets but from a context-sensitive collaboration that navigates tensions, fosters ownership, and embeds ecological recovery within economically and socially resilient landscape futures.

## Discussion and outlook

Policy efforts to halt biodiversity loss have been largely insufficient across Europe and globally. Despite decades of conservation initiatives, biodiversity continues to decline in many regions and across many taxa (Eichenberg et al. 2021; Kamp et al. 2021; Wirth et al. 2024). Evidence further suggests that protected areas have not been effective in mitigating this (Hallmann et al. 2017; Rada et al. 2019). In this light, Germany and the European Union exemplify many of the difficulties faced by multifunctional landscapes where agricultural intensification remains a dominant pressure on biodiversity (see e.g., Henle et al. 2008; Batáry et al. 2017), and the root causes of biodiversity decline are not yet adequately addressed by current European conservation policies (see, e.g., Pe’er et al. 2020).

Our study in the Oder Delta reveals a combination of key enablers and persistent obstacles to rewilding across ecological, economic, and social dimensions. The restoration of trophic complexity and ecological functions is already benefitting from natural recolonization processes originating in adjacent areas of Germany and Poland, likely facilitated by EU-wide protection regulations (Ledger et al. 2022) and by strong public support for ecological rewilding measures at the national level in Germany, despite lower levels of support and more differentiated perspectives among the local population in the Oder Delta (Dunn-Capper et al. 2024; Massenberg 2025). Together with local stakeholder support for restoring hydrological connectivity, these conditions create a favorable context for the recovery of more ecologically integral, self-regulating ecosystems that can deliver positive biodiversity outcomes and reduce risks to society (i.e., flood and disease regulation). Societal enablers further include the presence of trusted regional NGOs, the use of participatory and dialogue-oriented approaches, and deliberate efforts to foster mutual learning and long-term relationships among diverse actors. These factors support cooperation and help embed rewilding initiatives within local place identities. From an economic perspective, the integration of rewilding into regional economic development remains an important frontier. The enterprises surveyed in the Oder Delta expressed optimism about the potential economic benefits of a “wilder landscape”, particularly in tourism and regional branding. Creating a cross-border regional brand, such as “Oder Delta”, could align tourism and sustainable development goals while promoting nature conservation. At the same time, significant obstacles remain, including landscape fragmentation and new barriers to species dispersal—such as large-scale fencing infrastructure—that may hinder further ecological recovery, disrupted flood regimes, and the legacy of historical land-use in floodplains and forests. Limited public awareness of rewilding, skepticism rooted in past conflicts, fragmented governance structures, and competing land-use interests can further complicate support for rewilding actions. In parallel the fragmentation of tourism promotion (see “Economic dimension” section) highlights the need for better cross-sectoral coordination to align economic and ecological goals. Addressing these obstacles requires adaptive rewilding planning sustained by ecological monitoring, societal engagement, coordination across governance levels, and a long-term commitment to inclusive and adaptive processes.

The contribution of rewilding to the future conservation debate lies in its ability to address multiple ecological processes and socio-economic goals simultaneously. In the face of the ongoing biodiversity crisis, established restoration approaches will increasingly need to prioritize limited resources across multiple conservation needs. Rewilding principles can inform these difficult decisions by offering a broader perspective that extends beyond simpler ecological criteria, such as red list status (see, e.g., Carver et al. 2021). At the same time, the debate regarding the relationship between rewilding and ecological restoration continues (Carver et al. 2025). In the Oder Delta, the rewilding approach and opportunities identified here align with ecological restoration perspectives and selected associated principles, such as stakeholder engagement and collaborative knowledge production (Gann et al. 2019), yet placing particular emphasis on process-oriented restoration and self-sustained ecosystems (Carver et al. 2021).

Rewilding is increasingly adopted as an ecological restoration framework by conservation initiatives, particularly in Europe (Jepson et al. 2018; Root-Bernstein et al. 2018), focusing on the integral recovery of ecological processes and landscape-wide resilience. Beyond benefits to biodiversity, rewilding has significant potential to promote a more integrative approach to landscape management, recognizing the diverse societal needs tied to landscapes. Rewilding projects should emphasize the importance of a holistic vision of landscape management reconciling ecosystem conservation objectives with demands from sectors like water, agriculture, energy, and infrastructure. In the European context, landscapes undergoing rewilding are rarely managed solely for biodiversity restoration, as they must also address sustainable land use strategies and novel resource-use practices (Rey Benayas et al. 2025). The Oder Delta is an example where rewilding options are being explored despite intense land use and strong public perception of human–wildlife conflicts as major barriers (Fig. 1). At the same time, growing stakeholder awareness of the environmental problems—particularly past floodplain transformation and overexploitation of water resources—is enabling large-scale restoration projects.

The integration of participatory processes plays a crucial role in gaining support for rewilding and achieving successful rewilding outcomes (Quintero-Uribe et al. 2022). Public support for rewilding is highly dependent on transparent, inclusive dialogues that mediate between diverse stakeholder interests. As observed in Rothenklempenow in the Oder Delta, local initiatives that focus on collaborative goal-setting and scenario development may help identify relevant rewilding pathways while considering the broader socio-economic context. These processes, by facilitating dialogue and shared decision-making, help bridge the gap between ecological objectives and economic realities, ensuring that rewilding becomes a community-driven effort that integrates diverse local values and needs (Reed et al. 2013; Ferraro and Whitehead 2025). Regarding rewilding in practice, moving beyond the concept of “wilderness” is crucial for ensuring rewilding’s broader acceptance. While “wilderness” often evokes a preservationist mindset, rewilding should be understood as a dynamic, evolving process (see, e.g., Jørgensen 2015; Perino et al. 2019; Massenberg et al. 2023). Small individual contributions, facilitated by participatory processes, can play a significant role in reshaping landscapes, contributing to the resilience of social-ecological systems. These actions, though small at the individual level, collectively support the broader goal of restoring ecological functions, improving connectivity, and enhancing ecosystem services. On a conceptual level, scientifically sound and consistent definitions of rewilding are vital for clarifying the concept and dispelling confusion, particularly regarding the notion of wilderness.

Rewilding projects often take place in local contexts where stakeholders may not share the same linguistic or conceptual understandings of rewilding. Translations cannot easily reflect the subtle nuances of meaning in academic debate and the use of specific terms can sometimes generate incomprehension or even resistance. Rewilding perspectives differ significantly between regions. In North America, the wilderness-perspective has been prominent, while in Europe the focus may also lie on wildness. Studies from Norway (Engen et al. 2018), Spain (Schmitz et al. 2021), and the UK (Wynne-Jones et al. 2018) show that rewilding’s legitimacy depends heavily on how it is framed, e.g. a restoration approach that focuses on the practical use of natural resources versus narratives centered around preserving untouched nature or the emerging conservation concept of rewilding.

The Oder Delta case illustrates how rewilding can complement existing restoration approaches when embedded in long-term regional collaboration. Yet, such approaches are resource-intensive. Stakeholder meetings, excursions, and cultural events require time and institutional commitment, conditions that were met in this case due to over a decade of local engagement by ROD. Transferring this model elsewhere means recognizing the investment needed to build legitimacy and shared ownership to shape rewilding as a long-term, socially embedded alternative or supplement to established restoration approaches such as the designation of protected areas.

Ultimately, rewilding’s potential lies in its ability to connect ecological restoration with local realities. Realizing this potential will require navigating fragmented policy frameworks, fostering cross-sectoral integration (e.g. biodiversity and climate policies, agriculture, renewable energy production, infrastructure development, nature-based tourism and water resource management), and engaging stakeholders early and meaningfully to resonate with local values, foster broader acceptance and mobilize active support. When approached holistically, rewilding not only promises restoring ecological functions but can also provide a platform for addressing the diverse needs of society, laying the foundation for resilient landscapes that meet positive socio-ecological interactions.

## Footnotes

1 https://rewildingeurope.com/
